# Albumin losses during hemodiafiltration: all dialyzers are not created equal - a case report

**DOI:** 10.1186/s12882-019-1567-8

**Published:** 2019-10-28

**Authors:** Charles Cuvelier, Michel Tintillier, Gabriela Migali, Charlotte Van Ende, Jean-Michel Pochet

**Affiliations:** CHU UCL Namur, Internal Medicine and Nephrology Departement, Université catholique de Louvain, Sainte-Elisabeth site, 15 Place Louise Godin, Namur, Belgium

**Keywords:** Online hemodiafiltration, High permeability membrane, Convective volume, Hypoalbuminemia, Albumin loss, Polyphenylene membrane, Phylther SD

## Abstract

**Background:**

Online hemodiafiltration (OL-HDF) is associated with better removal of both small and middle molecules and might improve survival compared to conventional hemodialysis (HD). Nevertheless, hemodiafiltration (HDF) can lead to an increase in albumin loss across the dialyzer, especially with high permeability membrane and high convective volume (CV). We present the case of a patient treated by OL-HDF who developed severe hypoalbuminemia resulting from massive albumin loss into dialysate.

**Case presentation:**

A 71-year-old woman with ESRD started renal replacement therapy in December 2016. She was treated by high volume post-dilution OL-HDF, 4 h, 3 times per week. The dialyzer was the Phylther HF20SD (a 2.0m^2^ heat sterilized high flux (HF) polyphenylene membrane from Bellco). At the initiation of dialysis, the serum albumin was 4.0 g/dl. During the following months, the patient developed severe hypoalbuminemia. The lowest value observed was 2.26 g/dl in July 2017. Diagnostic workup excluded nephrotic syndrome, hepatic failure and malabsorption. The patient was shifted from OL-HDF to standard HF HD, keeping the same dialyzer and dialysis schedule. During the following months, we observed a progressive correction of the hypoalbuminemia (3.82 g/dl at last follow-up). To precise the impact of the epuration technique on the albumin losses in this patient, we measured the amount of albumin in dialysate during one session with the Phylther HF20SD on OL-HDF and one session with the same filter but on standard HD. The CV was 29.0 l for the HDF session. The total albumin losses were 23.6 g on OL-HDF and 4.6 g on HD.

**Conclusion:**

OL-HDF can lead to significant albumin loss into the dialysate, especially with high permeability membrane and high CV. When prescribing post-dilutional OL-HDF, the choice of the dialyzer membrane should be made with caution. Users of the steam sterilized polyphenylene membrane, the Phylther SD, should be informed of the risk of large albumin loss with this membrane during post-dilution OL-HDF.

## Background

Hemodiafiltration (HDF) provides better clearance of medium-high molecular weight solutes than conventional hemodialysis (HD), by a combination of diffusive and convective solutes transport through a high flux (HF) membrane [1] and might improve survival [2–4]. However, online hemodiafiltration (OL-HDF) can lead to an increase in albumin loss across the dialyzer, especially with high permeability membrane and high convective volume (CV) [5, 6]. We report the case of a 71-year old woman who developed severe hypoalbuminemia resulting from massive albumin loss during dialysis while treated by high-volume post-dilutional OL-HDF with a large surface steam sterilized polyphenylene HF dialyzer, the Phylther HF20SD (Bellco). We will discuss the issue of albumin leakage through the hemodialysis membrane with different extracorporeal dialysis modalities and argue against the use of this specific membrane, the Phylther SD, for OL-HDF.

## Case presentation

This 71-year-old woman with ESRD related to chronic interstitial nephritis started renal replacement therapy in December 2016. Her past medical history included high blood pressure, paroxysmal junctional tachycardia, peptic esophagitis and multinodular goiter.

She was treated from the beginning by post-dilution OL-HDF through a left arm fistula, 4 h, 3 times per week. Her dry weight was around 62 kg. The dialysis monitor was a 5008 Cordiax equipped with ‘AutoSub plus’ (Fresenius Medical Care) to maximize HDF substitution volume which was usually 28–30 l per session. The dialyzer was a Phylther HF20SD (a 2.0 m^2^ heat sterilized HF polyphenylene membrane from Bellco).

**Fig. 1:**
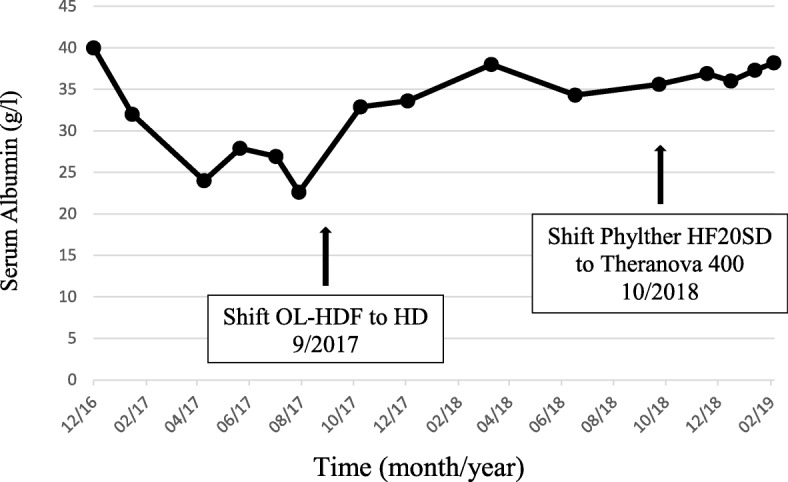
Evolution of Albuminemia (g/l) over time. Vertical Axis: Serum Albumin (g/l). Horizontal Axis: Time (month/year)

At the initiation of dialysis, total serum protein and albumin were 6.0 g/dl and 4.0 g/dl, respectively. Urine albumin was 0.38 g/l, 3 months before starting dialysis. During the following months, the patient developed severe hypoalbuminemia. The lowest value observed was 2.26 g/dl in July 2017 (see Fig. 1.). At that time, albuminuria was measured at 105 mg/24 h. There was no symptom of malabsorption. A 72-h stool collection show no steatorrhea. Duodenal biopsies were normal. There was no sign of liver failure.

We hypothesized that hypoalbuminemia might result from albumin loss across the dialyzer. In September 2017, the patient was shifted from OL-HDF to standard HF HD, keeping the same dialyzer and dialysis schedule. During the following months, we observed a progressive correction of the hypoalbuminemia as illustrated in Fig. 1.

In October 2018, we shifted the patient on a medium cut-off (MCO) filter, the Theranova 400 (a 1.7 m^2^ polyarylethersulfone – polyvinylpyrrolidone membrane from Baxter). The albuminemia remained stable.

To better delineate the impact of the epuration technique on the albumin losses we measured recently in this patient the amount of albumin in the total dialysate collection during one session with the Phylther HF20SD on OL-HDF and one session with the same filter but on standard HD. The convective volume was 29.0 l for the HDF session. The total albumin losses were 23.6 g on OL-HDF and 4.6 g on HD.

## Discussion and conclusions

The recognition the potential toxicity of several medium-high molecular weight solutes accumulating in ESRD patients has encouraged the development of more permeable dialysis membranes and the popularization of HDF, especially in Europe [7]. Indeed, if the removal of middle-sized molecules, such as β_2_-microglobulin (β_2_m), is increased with HF membranes, their performance can be markedly enhanced by the addition of convection through HDF [1].

Three recent randomized controlled studies comparing HDF to conventional low flux [2] or HF HD [3, 4] suggested that OL-HDF might improve survival when providing high convective volumes. Indeed, secondary post hoc analysis of the CONTRAST [2] and Turkish HDF Study [3] showed a survival benefit in the patient group achieving the highest CV. The ESHOL Study only retained for analysis the patients able to reach a high reinfusion volume (> 18 l) and demonstrated a 30% reduction of all-cause mortality in the HDF group [4]. Interestingly, the mean delivered CV was 23.7 l /session in the ESHOL study which was somewhat higher than the average volumes reached in the aforementioned trials [2–4]. These results suggest that post-dilutional HDF could modify patient survival when a sufficient CV is reached. A minimum replacement volume of 21 l or total CV of 23 l per session have been recommended [4, 8].

Among the prerequisites to perform successful high-volume HDF are a high blood flow, a dialysis machine with automated replacement volume and an appropriate dialyzer. Modern HF dialyzers, suitable for high-volume HDF, require a high ultrafiltration coefficient, greater than 20 ml/h/mmHg/m^2^, and a high clearance of medium and large-sized molecules, usually defined by sieving coefficient (SC) for β_2_m greater than 0.6 [1, 9]. One issue related to membranes with larger pores to remove molecules in the range of β_2_m is the potential loss of albumin. Indeed, if albumin losses are usually absent or low with HF HD (between 0 and 2 g/4 h treatment), albumin losses may be greater in HDF with the same membrane, especially in the post-dilution mode and with higher CV. This has been well demonstrated in a study who evaluate 3 patients treated with 8 different HF dialyzers in post-dilution HDF at variable ultrafiltration/substitution rate (0, 30, 60 and 90 ml/min) [5]. The albumin loss per session increased markedly, from < 2 g at a filtration rate of 30 ml/min to up to 7 g at a filtration rate of 90 ml/min. There was also a wide discrepancy between the different dialyzers with albumin losses ranging from 0.3 g/4 h to 7 g/4 h at the maximal filtration rate.

In fact, not all HF dialyzers are suitable to perform OL-HDF*.* A recent study examined the efficiency and safety characteristics of 19 dialyzers in such condition. The authors found that 6 dialyzers were associated with albumin loss > 5 g per session, a cut-off above which they were considered not suitable for OL-HD [10]. The most notable albumin losses (up to 17 g/session) were observed with the steam polyphenylene membrane, the Phylther HF22SD (Bellco), the one (in 2.0 m^2^) used in our patient who lost nearly 24 g albumin/4 h treatment. These major albumin losses contrasted with the limited albumin losses (< 3 g/session) observed with the gamma rays release of the same membrane, the Phylther HF22F. This discrepancy suggests that high thermal stress during processing might impact the original size of the pores and increase the membrane permeability [11]. Those results also contrast with reassuring in vitro data, more specifically the SC of albumin of 0,003 reported in the dialyzer Phylther SD data sheet. However, Hulko M. et al. showed that testing conditions have a marked impact on the measurement of SC and found no good correlation between in vitro measured coefficient values and in vivo reported clinical albumin loss in HDF mode [12].

Hypalbuminemia is frequent in ESRD and has been associated with increased morbidity and mortality in patients on maintenance HD [13]. Albumin levels depend on the rate of albumin synthesis by the liver, the albumin fractional catabolic rate (FCR) and its distribution between the vascular and extravascular space. Poor nutrition is a common cause of hypoalbuminemia as inadequate protein and calorie intake decrease albumin synthesis. The predominant cause of hypoalbuminemia in the dialysis population is inflammation as the acute phase response inhibits albumin synthesis and increases FCR, leading to declining serum albumin [14]. The combination of inflammation and malnutrition might lead to more severe hypoalbuminemia and the malnutrition, inflammation, atherosclerosis (MIA) syndrome [15]. External albumin losses such as renal losses (proteinuria), enteral losses and even transmembrane loss during dialysis might also contribute to the development of hypoalbuminemia [14], as illustrated by the case presented here.

Whether excessive albumin removal during dialysis treatment is actually harmful is a question of greater interest as modern dialyzers with high permeability expose the patient to a greater risk of albumin loss. Compared to traditional HF membranes, such membranes, referred as protein leaking membranes (PLM), super-flux (SF) membranes or high-performance membranes (HPMs), improved the clearance of large low molecular weight proteins (LMWPs) and highly protein bound uremic toxins, like proinflammatory cytokines, complement factor D, α_1−_microglobulin but at the cost of increased albumin losses ranging from 2 to 6 g/HD treatment [14, 16–20]. The acceptable limit of albumin losses during dialysis remained to be established. Available data suggest that the routine use of dialyzers resulting in a weekly loss of < 12 g/week appears to pose little risk [21, 22]. Maduell F. recommend avoiding membranes with albumin losses greater than 5 g/session when describing the prerequisites of successful high-volume HDF [9]. Obviously, the heat sterilized Phylther SD does not fulfil this condition.

Very recently, a new generation of promising HD dialyzers, called MCO or high retention onset dialyzers, has been developed. The concept is that both cut-off and retention onset values are close to each other’s with a steeper sieving curve and a cut-off value lower than that of albumin. Thanks to their enhanced permeability and selectivity, those MCO membrane allow an increased removal of middle-to-high weight range uremic toxins while limiting albumin loss to an acceptable level. The use of these membranes in the HD mode does not necessitate special equipment nor increased water consumption and is known as expanded HD [23]. In 2 pilot studies, 3 MCO dialyzers prototypes, including the Theranova 400 (Baxter), were compared to HD and OL-HD with last-generation HF dialyzers (FX Cordiax 80 and FX Cordiax 800 (Fresenius Medical Care)). MCO HD provides more efficient clearance of larger middle molecules than HF HD and even than OL-HDF from some of them, like free light chains (FLC). In the second study, albumin removal with Theranova 400 (median 3.2 g (range 1.9–3.9)) was greater compared to HF-HD (0.2 g (0.2–0.3)) and OL-HDF (0.4 g (0.3–0.8)) but not excessive [24]. Interestingly, a study compared the Theranova 400 with 4 different modern HF dialyzers of comparable surface (the Phylther, the Revaclear 400 (Baxter), the FX Cordiax 80, and the Solacea (Nipro)) in HD mode [25]. The best performing dialyzers in term of removal of different toxins (β_2_m, Myoglobin, FLC) were the Phylther and the Theranova 400. The authors concluded that the Phylther might be comparable to the new MCO dialyzer Theranova 400 regarding removal of middle high size uremic toxins. Surprisingly, albumin losses/session were substantially more important with the Phylther (4.1 g) than with the Theranova 400 (1.9 g) and the other membranes (0.26 to 1.53 g). Similar results were obtained in a recent prospective study from Maduell et al. comparing 4 dialyzers, including the Phylther 17SD, the FX80 Cordiax and the Theranova 400. Albumin losses/session were significantly higher with the Phylther in HD than with the FX 80 in HD, the FX 80 in OL-HDF and the Theranova 400. The authors conclude that steam sterilized polyphenylene membrane should be used in HD or eventually in pre-dilution OL-HDF, but should not be prescribed for post-dilutional OL-HDF [11]. Of note, the manufacturer restricts the use of the MCO membrane Theranova to HD, at variance with the manufacturer of the steam polyphenylene Phylther SD who promote the use of his dialyzer for both HD and HDF.

In conclusion, the current case illustrates that OL-HDF can lead to significant albumin loss into the dialysate and cause hypoalbuminemia, especially with high convective volume and high permeability membrane, as the Phylther SD membrane. Indeed, despite in vitro characteristics of a traditional HF dialyzer, this polyphenylene membrane behaves like a PLM in vivo. When prescribing post-dilutional OL-HDF, the choice of the dialyzer membrane should be made with caution. Users of the steam sterilized polyphenylene membrane, the Phylther SD, should be informed of the risk of large albumin loss with this membrane during post-dilution OL-HDF.

## Data Availability

All data generated or analyzed are included in this published article.

## Abbreviations

CV: Convective volume
FCR: Fractional catabolic rate
FLC: Free light chains
HD: Hemodialysis
HDF: Hemodiafiltration
HF: High flux
HPMs: High-performance membranes
LMWPs: Low molecular weight proteins
MCO: Medium cut-off
OL-HDF: Online hemodiafiltration
PLM: Protein leaking membrane
SC: Sieving coefficient
SF: Super-flux
